# Dynamic Imbalance Analysis and Stability Control of Galloping Gait for a Passive Quadruped Robot

**DOI:** 10.1155/2015/479615

**Published:** 2015-06-21

**Authors:** Chunlei Wang, Ting Zhang, Xiaohui Wei, Yongjun Long, Shigang Wang

**Affiliations:** ^1^The State Key Laboratory of Mechanical System and Vibration, Shanghai Jiao Tong University, Shanghai 200240, China; ^2^College of Mechanical Engineering, Shanghai University of Engineering Science, Shanghai 200030, China

## Abstract

Some imbalance and balance postures of a passive quadruped robot with a simplified mathematical model are studied. Through analyzing the influence of the touchdown angle of the rear leg on the posture of the trunk during the flight phase, the stability criterion is concluded: the closer are the two moments which are the zero time of the pitching angle and the peak time of the center of mass, the better is the stability of the trunk posture during the flight phase. Additionally, the validity of the stability criterion is verified for the cat, greyhound, lion, racehorse, basset hound, and giraffe. Furthermore, the stability criterion is also applicable when the center of the mass of body is shifted. Based on the stability criterion, the necessary and sufficient condition of the galloping stability for the quadruped robot is proposed to attain a controlled thrust. The control strategy is designed by an optimization dichotomy algorithm for seeking the zero point of the balance condition. Through the control results, it is demonstrated that the imbalance posture of the trunk could be stabilized by adjusting the stiffness of four legs.

## 1. Introduction

In recent years, some models were presented to study the trotting, bounding, and galloping gait of quadruped robot. The spring loaded inverted pendulum (SLIP) model was suitable to study the hopping of one-legged robot [1, 2], bipedal running of human [3–5], and trotting of quadruped robot [6, 7]. Meanwhile, the studying of SLIP model was a fundamental work for other models of the quadruped robot. The planar model consisted of a rigid body and four springy massless legs was derived from the SLIP [8]. Many bounding [9–12] and galloping [7, 13] gaits were achieved in simulation or experiment environments based on the beam and springy leg (BSL) model.

The stability was an important property of locomotion, since the stability could ensure the quadruped robot form a successive locomotion. Most of the authors studied the motion stability of quadruped robot by employing the Poincaré return map (PRM) based on the Newton-Raphson method [10, 11, 14–16]. The PRM method was designed to search for a group of initial values for maintaining a stable cyclic motion through solving the dynamic equation of the quadruped robot. If the selected initial values were not able to maintain a steady-state bounding or galloping, another group of initial values was used to solve the dynamic equation to judge whether the locomotion was stable or not. The procedures above were repeated until the desired stable locomotion was achieved. Ultimately, many stable cyclic motions would be achieved by the same mechanical parameters of robot. Due to the difficulty in hand-tuning multiple parameters, the optimization algorithm was chosen to search for the initial values when the system was passive [14, 17] or was used to design the control parameters when the system was controlled [18–20]. From the analysis of the PRM method, the passive dynamic bounding and galloping gait were achieved by cut and try. The necessary and sufficient conditions of the stability during the stride were not concerned when using the PRM method. Therefore, the dynamic stability inside the stride was an elusive issue for being not studied elaborately.

Meanwhile, other authors also focused on achieving the stable bounding or galloping gait by control strategies. Marhefka et al. [13] presented a fuzzy control strategy for the high-speed quadruped bounding and galloping. The fuzzy logic controller was able to search for the touchdown angles and the thrusts of legs by tracking the desired running height and the desired velocity of a quadruped robot in one stride. Finally, the galloping gait was stabilized by the fuzzy logic controller with the planar BSL model. Krasny and Orin [18] realized a 3D galloping gait of a small dog with the finite state machine. The controller adjusted the rest position of leg spring to match the designed energy of each leg when it was at the maximum compression. The control algorithm could stabilize the pitching motion and the vertical displacement of center of mass (COM) which were considered to be the two key indicators of the stability in the sagittal plane. Seyfarth et al. [21] presented a movement criterion for running. They concluded that the mechanical self-stabilized running would be achieved by adjusting the leg stiffness for a certain attack angle. At last, the stable running was realized for a SLIP model by the PRM method. Herr and McMahon [22] employed a ‘hip thrusting and shoulder braking' method to ensure the pitching stability and realized a stable galloping gait of a horse. Designing a control strategy could attain stable running including the trotting, bounding, and galloping gait. However, the dynamic balance conditions were not analyzed elaborately. Hence, a necessary and sufficient condition must be proposed for judging the stability of the quadruped robot.

As stated in previous paragraph, the stability indicators in the sagittal plane are the pitching angle and the height of COM. Meanwhile, the pitching angular velocity of the trunk during the flight phase also plays an important role in maintaining stable locomotion of the robot. In this paper, the instable state is defined as the tumble of robot; that is, the bottom or the head falls down on the ground while the quadruped robot is galloping. Through the BSL model, the imbalance postures of the robot with improper pitching angular velocity are studied in this paper. Then, the stability criterion is achieved. Next, the controlled thrust is designed by adjusting the leg stiffness on the basis of the stability criterion. Finally, through some simulations, it is demonstrated that the control method is feasible to stabilize the trunk posture under the instable initial parameter while the quadruped robot is galloping.

In Methods, the simplified model is introduced and the controlled thrust is proposed. In Results, by analyzing the impacts of the pitching angle and angular velocity on the trunk posture, the stability criterion is proposed. Meanwhile, the controlled thrusts are obtained to stabilize the locomotion by adjusting the leg stiffness. In Discussions, the validity of the stability criterion is verified for various animals. Finally, in Conclusions, some summaries are given.

## 2. Methods

### 2.1. Dynamical Model and Galloping Simulation of a Simplified Quadruped Robot

**Figure 1:**
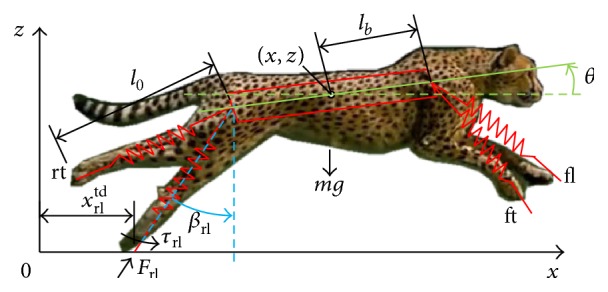
The simplified quadruped model of a cheetah.

In the paper, a quadruped robot is used for the purpose of analyzing the stability of the galloping gait and is simplified as a BSL model in sagittal plane [6, 12], as shown in Figure 1. The COM coordinate is (*x*, *z*), the body pitching angle is *θ*, and the leg orientation angle is *β* while the quadruped robot is running. In addition, the four legs (front leading leg, front trailing leg, rear leading leg, and rear trailing leg) are indicted as fl, ft, rl, and rt, respectively. Furthermore, based on the energy conservation and the Lagrange equations, the dynamic model of the BSL system is constructed when the damping energy dissipation terms are ignored.

The dynamic model is derived as

(1) $$\begin{array}{c} M\ddot{q}+B\left(\;q\;\right)=Q\left(\;q\;\right), \end{array}$$

where $M=\left[\begin{array}{ccc} m & 0 & 0\\ 0 & m & 0\\ 0 & 0 & J \end{array}\right]$, *q* = [*x* *z* *θ*]^*T*^. In addition,  *B*(*q*) = [*B*_1_(*x*, *z*, *θ*) *B*_2_(*x*, *z*, *θ*) *B*_3_(*x*, *z*, *θ*)]^*T*^,

(2) B1x,z,θ=Kδftl0−lftsin⁡βft+δfll0−lflsin⁡βfl+δrtl0−lrtsin⁡βrt+δrll0−lrlsin⁡βrl,B2x,z,θ=−Kδftl0−lftcos⁡βft+δfll0−lflcos⁡βfl+δrtl0−lrtcos⁡βrt+δrll0−lrlcos⁡βrl+mg,B3x,z,θ=−Klbδftl0−lftcos⁡θ−βft+δfll0−lflcos⁡θ−βfl−δrtl0−lrtcos⁡θ−βrt−δrll0−lrlcos⁡θ−βrl.

Furthermore, *Q*(*q*) = [*Q*_1_(*x*, *z*, *θ*) *Q*_2_(*x*, *z*, *θ*) *Q*_3_(*x*, *z*, *θ*)]^*T*^,

(3) Q1x,z,θ=−δftFftsin⁡βft−δflFflsin⁡βfl−δrtFrtsin⁡βrt−δrlFrlsin⁡βrl+δftτftcos⁡βftlft+δflτflcos⁡βfllfl+δrtτrt·cos⁡βrtlrt+δrlτrlcos⁡βrllrl,Q2x,z,θ=−δftFftcos⁡βft−δflFflcos⁡βfl−δrtFrt·cos⁡βrt−δrlFrlcos⁡βrl+δftτftsin⁡βftlft+δflτflsin⁡βfllfl+δrtτrtsin⁡βrtlrt+δrlτrlsin⁡βrllrl,Q3x,z,θ=lbδftFftcos⁡θ−βft+δflFflcos⁡θ−βfl−δrtFrtcos⁡θ−βrt−δrlFrlcos⁡θ−βrl+δftτft1−lbsin⁡θ−βftlft+δflτfl1−lbsin⁡θ−βfllfl+δrtτrt1−lbsin⁡θ−βrtlrt+δrlτrl1−lbsin⁡θ−βrllrl.

**Figure 2:**
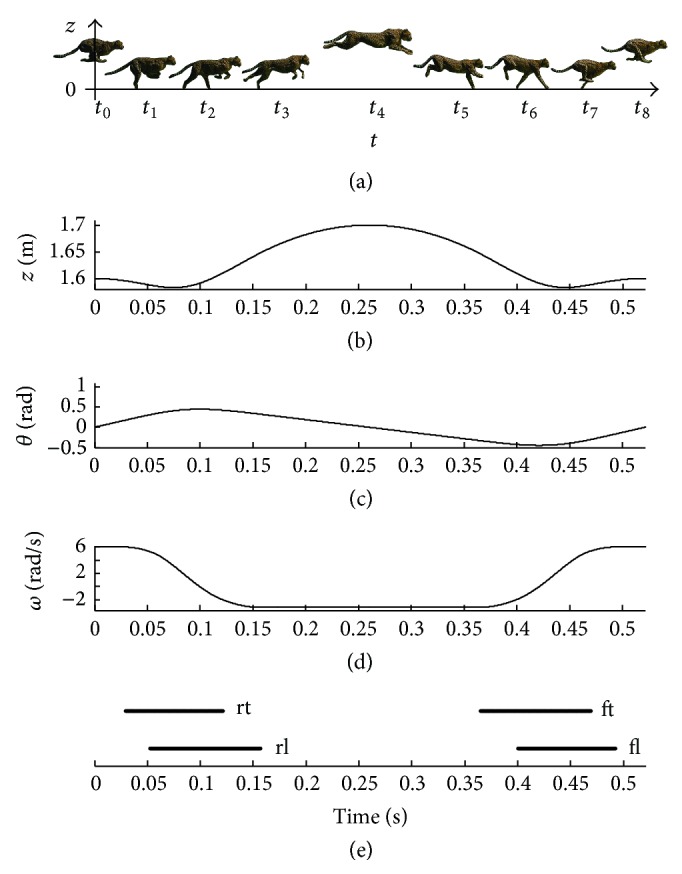
The galloping process of a cheetah.

Through the dynamic model Equation (1), Figure 2 shows the vertical displacement of the COM, the pitching angle *θ*, and the pitching angular velocity *ω* under the mechanical parameters and initial values in Table 1. The initial values include the horizontal displacement *x*_0_ and the vertical displacement *z*_0_ of COM, their velocities *v*_*x*0_ and *v*_*z*0_, the pitching angle *θ*_0_ and the pitching angular velocity *ω*_0_, and the orientation angles *β*_ft_^td^ and *β*_rt_^td^ at the moment of touchdown. The distance *d* between the leading and trailing leg when the legs are in contact with the ground is defined as follows:

(4) df=xfltd−xfttd,dr=xrltd−xrttd.

From Figure 2(a), a periodic galloping process of a cheetah is shown. At the moments *t*_0_ and *t*_8_, the spine of cheetah shows a maximum flexion posture and the COM of cheetah is at the peak in vertical direction. In addition, at the moments *t*_1_, *t*_2_, and *t*_3_, the rear legs touch down the ground, and the legs endure the ground reaction force (GRF). At the moment *t*_4_, the spine of cheetah shows a maximum extension when the COM of cheetah is at the peak in vertical direction and the pitching angle *θ* is zero. Furthermore, at the moments *t*_5_, *t*_6_, and *t*_7_, the front legs touch down the ground. Correspondingly, Figure 2(e) gives the footfall pattern and the stance duration of the four legs (fl, ft, rl, and rt). While one and two legs contact the ground during the stance phase, the velocity $\dot{q}(v_{x},v_{z},\omega )$ of the trunk is changing. On the contrary, during the flight phase, the legs do not exert the thrusts on the trunk, and, as a result, the velocity $\dot{q}(v_{x},v_{z},\omega )$ is constant.

However, practically, the trajectories of the galloping gait for the quadruped robot cannot be always a single kind of symmetrical locomotion. When the cheetah suffers from a disturbance, such as stepping on a loose stone, catching a small animal, or jumping over an obstacle, the cheetah then must keep the balance of the trunk for running forward. The trunk stability is dependent on the pitching angle *θ* and pitching angular velocity *ω*. In Results, the instability and stability analyses of the quadruped robot according to (1) are presented.

### 2.2. Control Design

**Figure 3:**
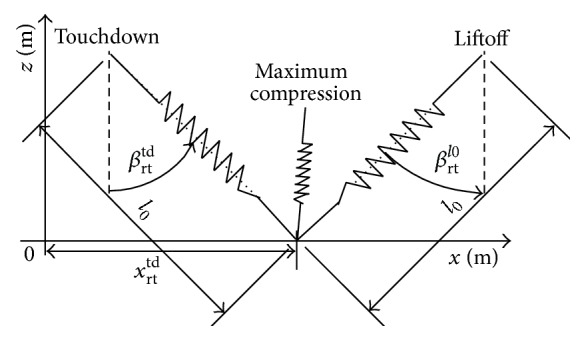
Stance phase of the rear tailing leg.

Based on the passive dynamic model [6, 14, 29], the mutual transformation of the kinetic energy and potential energy is widely studied. As shown in Figure 3, first, when the rear trailing leg touches down the ground, the leg starts retracting in a clockwise direction together with compression. Then, when the leg obtains the maximum compression, the elastic potential energy stored in the rear leg is the maximum. Finally, the stored elastic potential energy is released until the length of the leg reaches the rest length *l*_0_, and the leg starts to lift off the ground and the orientation angle is *β*_rt_^lo^.

**Figure 4:**
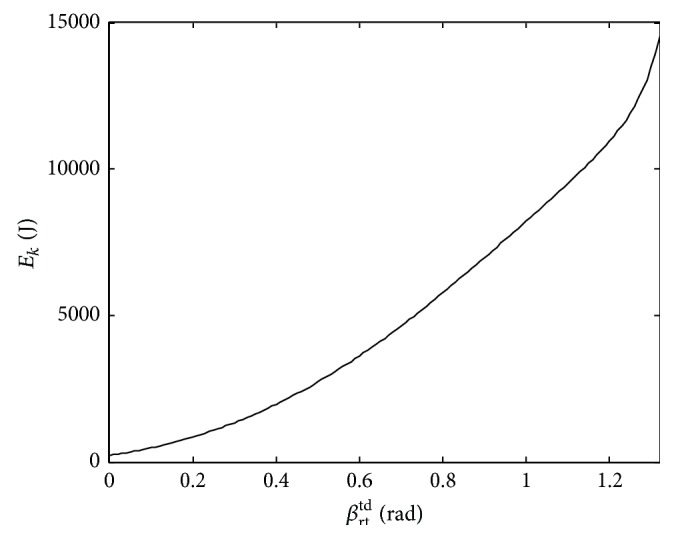
Maximum elastic potential energy stored in the rear legs.

In addition, the maximum elastic potential energy *E*_*k*_ stored in the two rear legs is shown in Figure 4 when *d*_*r*_ = 0 and the orientation angle *β*_rt_^td^ is increasing. Apparently, the elastic potential energy increases gradually and is expressed as

(5) $$\begin{array}{c} E_{k}=\max\left[\;\frac{1}{2}K{\left(\;l_{\text{rf}}-l_{0}\;\right)}^{2}+\frac{1}{2}K{\left(\;l_{\text{rt}}-l_{0}\;\right)}^{2}\;\right], \end{array}$$

where max⁡[·] is a maximum function.

From (5) and Figure 4, the stiffness of the legs could be adjusted to keep the energy constant when *β*_rt_^td^ is increased. As a result, the passive controlled thrusts are designed from the view of the mutual conversion of elastic potential energy, kinetic energy, and gravitational potential energy. The designed thrusts may be written as

(6) $$\begin{array}{c} F_{i}=\Delta K_{i}\left(\;l_{0}-l_{i}\;\right), \end{array}$$

where Δ*K*_*i*_ is the stiffness compensation achieved by an optimizing condition which will be discussed in Section 3.

During locomotion of the quadruped robot, the stiffness of legs has not been fully used to support the trunk. The leg stiffness is not constant but it can be adjusted by itself. When the robot wants to accelerate forward, the leg stiffness will decrease and when the robot wants to decelerate forward, the leg stiffness will increase. Therefore, in the paper, the stiffness compensation is thought as a passive control method [30] and plays an instruction role in active control [31] for the quadruped robot.

## 3. Results

### 3.1. Stability Analysis of the Quadruped Robot

**Figure 5:**
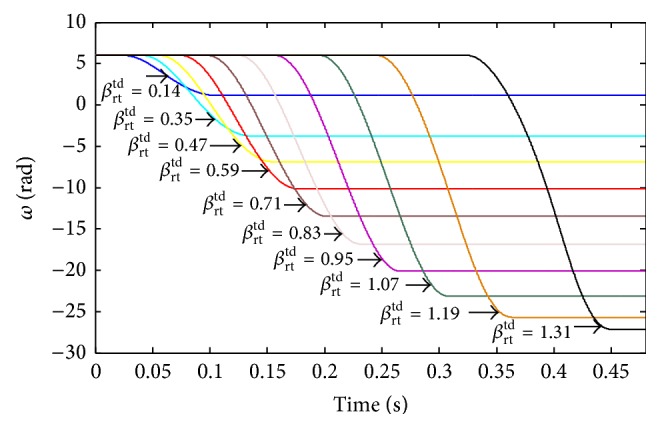
Pitching angular velocities at different touchdown angles.

**Figure 6:**
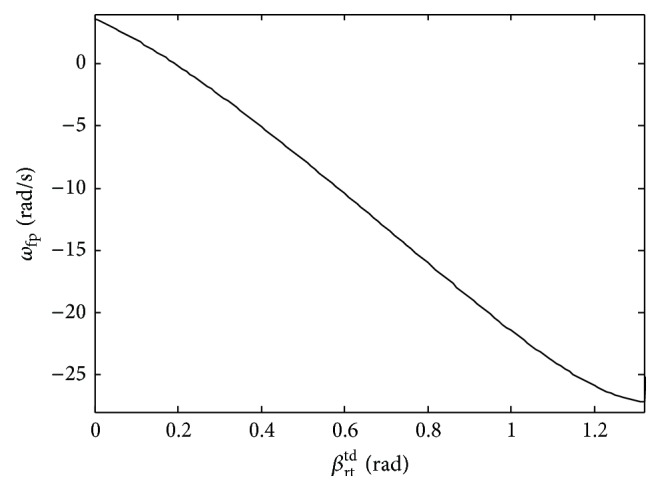
Pitching angular velocities during the flight phase.

For the passive quadruped robot, both the imbalance and balance postures depend on the initial mechanical parameters. When the touchdown angle *β*_rt_^td^ = (0 ~ *π*/2) rad and *d*_*r*_ = 0 under the initial values of the other parameters in Table 1, the trajectory *q*(*x*, *z*, *θ*) of the trunk may vary with different *β*_ft_^td^. As shown in Figure 5, the larger the touchdown angle *β*_rt_^td^ is, the larger, in the negative direction, the pitching angular velocity *ω*_fp_ of the trunk during the flight phase is. In addition, the pitching angular velocity *ω*_fp_ during the flight phase is given in Figure 6 under the different orientation angles *β*_rt_^td^. As shown, *ω*_fp_ increases approximately linearly when *β*_rt_^td^ = (0 ~ 1.2) rad. Next, the three states of the robot will be discussed with the different orientation angles *β*_rt_^td^.

**Figure 7:**
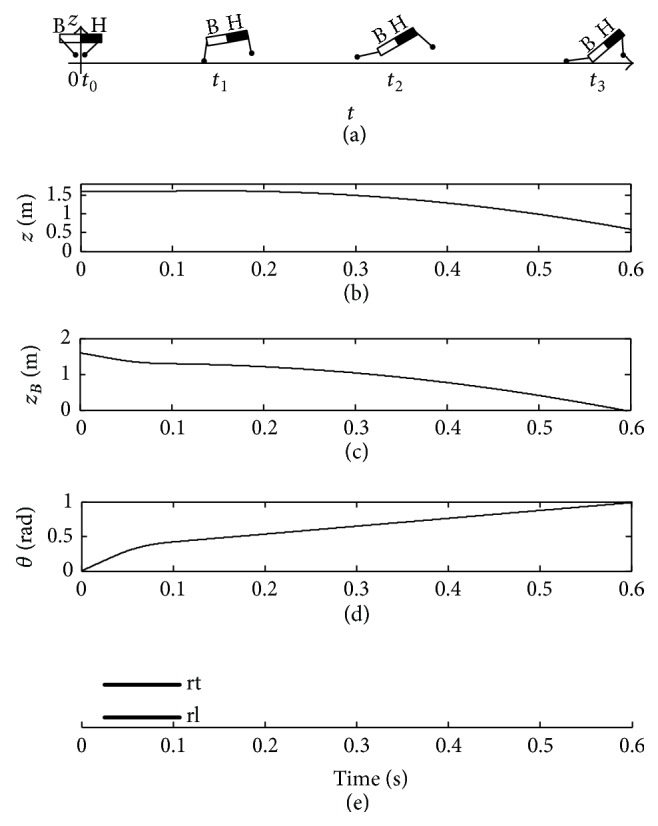
Imbalance posture of trunk with *β*_rt_^td^ = 0.14 rad.

Figure 7 shows the imbalance posture of the trunk with an insufficient pitching angular velocity. The black half body and white half body indicate “head” and “bottom” of the quadruped robot, respectively, in Figure 7(a). When the two rear legs touch down the ground at the same time under the orientation angle *β*_rt_^td^ = 0.14 rad, the pitching angular velocity *ω*_fp_ of the trunk is insufficient and the pitching angle *θ* is increasing in an anticlockwise direction after the legs lift off the ground in Figure 7(d). *z*_*B*_ is the vertical displacement of the bottom. In Figure 7(c), when *z*_*B*_ is zero, the bottom contacts the ground to make the quadruped robot fall down at the moment *t*_3_. Therefore, the insufficient touchdown angle *β*_rt_^td^ will lead to the insufficient pitching angular velocity *ω*_fp_ of the trunk. Consequently, the bottom first falls down on the ground in Figure 7(a).

**Figure 8:**
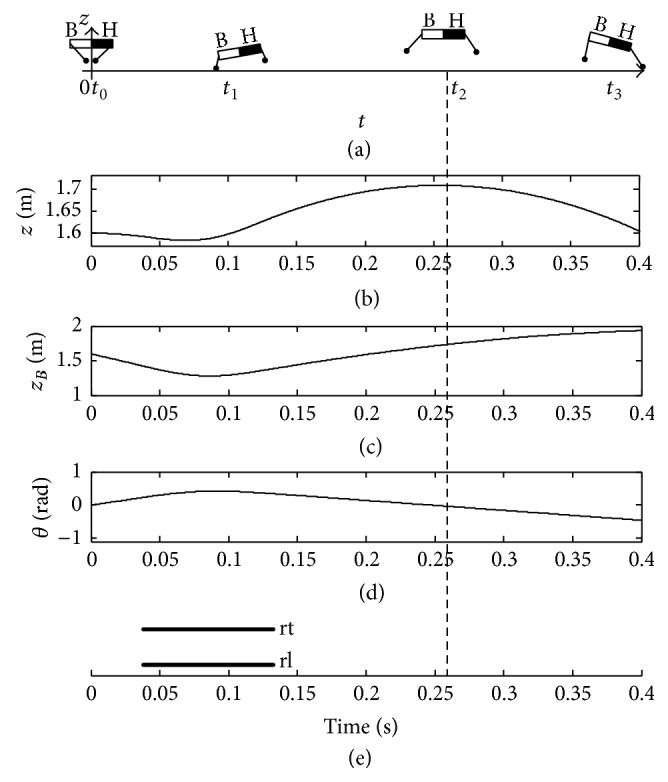
Balance posture of trunk with *β*_rt_^td^ = 0.32 rad.

Furthermore, when the initial touchdown angle *β*_rt_^td^ is equal to 0.32 rad, the pitching angular velocity *ω*_fp_ of the trunk is sufficient. As a result, the trunk shows a balance posture at the moment *t*_2_ in Figure 8. At the same time, the pitching angle *θ* is approximately zero at the peak time of COM in Figure 8(d). Consequently, the posture of the trunk is stable enough before it is supported by the two front legs at the moment *t*_3_ as shown in Figure 8(a).

**Figure 9:**
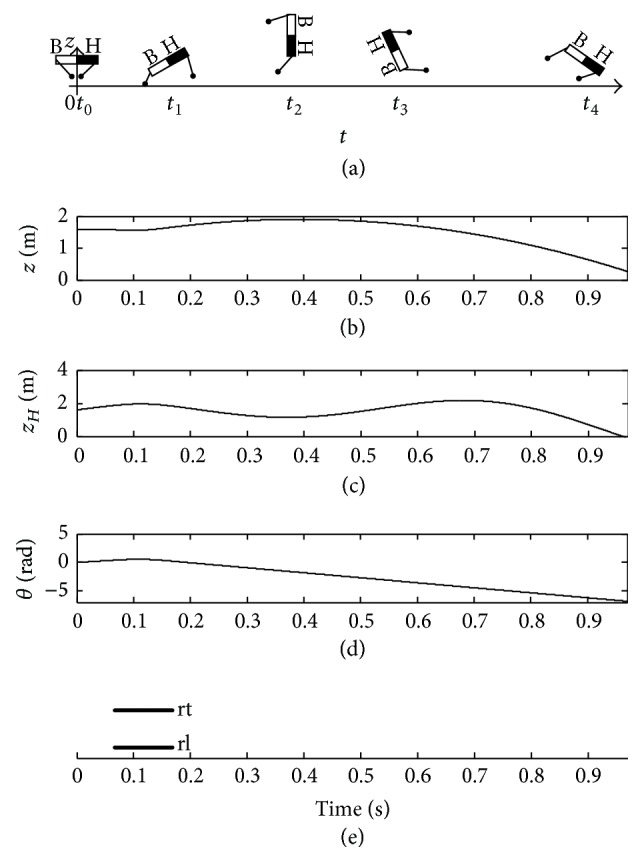
Imbalance posture of trunk with *β*_rt_^td^ = 0.54 rad.

When the initial touchdown angle *β*_rt_^td^ is equal to 0.54 rad, the pitching angular velocity *ω*_fp_ is overlarge. As shown in Figure 9(d), the pitching angle *θ* of the trunk is more than −*π*/2 rad at the moment *t*_2_ before the COM reaches the peak of vertical displacement. In addition, the pitching angle *θ* at the moment *t*_3_ is about −4.2 rad. Then, the head of the quadruped robot may fall down on the ground at the moment *t*_4_ as shown in Figure 9(a). *z*_*H*_ is the vertical displacement of the head shown in Figure 9(b). Therefore, the overlarge *ω*_fp_ will result in the pitching angle *θ* up to more than −*π*/2 rad before COM reaches the peak of the vertical displacement during the flight phase, so that the head, the bottom, or the back of the trunk may fall down according to the height of COM.

**Figure 10:**
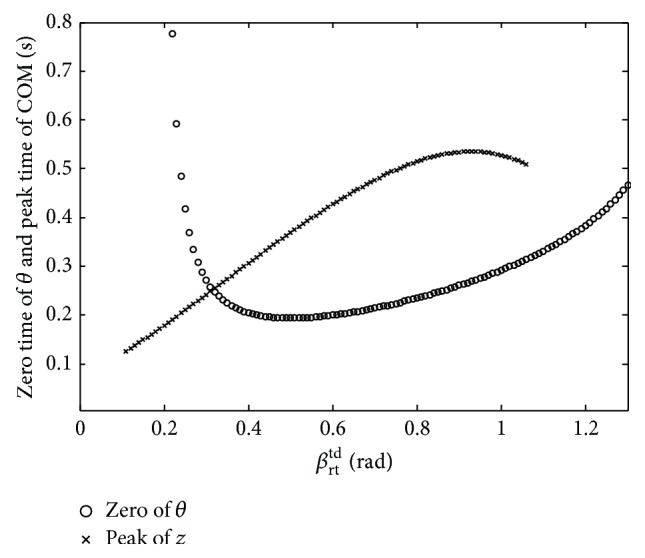
The zero time of pitching angle and the peak time of COM.

By analyzing the balance and imbalance postures of the trunk Figures 7~9, the initial condition *β*_rt_^td^ will affect the pitching angle *θ* and pitching angular velocity *ω*_fp_ during flight phase. Moreover, during *β*_rt_^td^ = (0 ~ *π*/2) rad, when the two moments, which are the zero time of the pitching angle *θ* and the peak time of COM during flight phase, occur at the same time, the trunk shows the best balance posture. In Figure 10, when the *β*_rt_^td^ increases from 0 to *π*/2 rad, the zero time curve of *θ* and peak time curve of COM are given. As is shown, when *β*_rt_^td^ = 0.32 rad and the moment is about 0.25 s, the pitching angle *θ* is zero. At the same time, the COM reaches the peak of vertical displacement. When *β*_rt_^td^ is far less than 0.32 rad, the bottom of the quadruped robot will fall down on the ground. When the *β*_rt_^td^ is far higher than 0.32 rad, the pitching angle *θ* of the trunk will be negatively more than −*π*/2 rad during the flight phase. In other words, the closer are the two moments which are the zero time of the pitching angle *θ* and the peak time of COM, the better is the stability of the trunk posture during the flight phase.

### 3.2. Control Result for the Instability of the Quadruped Robot

*v*_*z*_^lo^, *θ* ^lo^, and *ω* ^lo^ indicate the vertical velocity, pitching angle, and angular velocity of the quadruped robot when rear leading leg lifts off the ground and the locomotion of the COM is described as

(7) Δzp=vzlo·tp−12g·tp2,0=vzlo−g·tp,θlo=0−ωlo·tω,12m·vzlo2=mg·Δzp,

where Δ*z*_*p*_ is the vertical displacement of COM from liftoff to the peak during the flight phase, *t*_*p*_ is the corresponding time interval, *g* is the acceleration of gravity, and *t*_*ω*_ is the time interval from liftoff to the zero point of *θ* during the flight phase.

Based on the discussion of Figure 10, if *t*_*p*_ = *t*_*ω*_, a best balance posture of the trunk during the flight phase is presented. Therefore, (7) may be transformed as

(8) $$\begin{array}{c} \text{Con}=\omega^{\text{lo}}v_{z}^{\text{lo}}+g\theta^{\text{l}o}=0. \end{array}$$

In a word, (8) is the necessary and sufficient condition of a best balance for the quadruped galloping. When the quadruped robot exposes an imbalance posture with a random touchdown angle *β*_rt_^td^, the controlled thrusts equation (6) is designed to compensate the difference between the zero time of pitching angle and the peak time of COM by solving the best balance condition equation (8) and the dynamic equation (1) coordinately. Then, the controlled thrust which depends on the coefficient Δ*K*_*i*_ in (6) is achieved by using an optimization algorithm to find zero point of (8).

**Figure 11:**
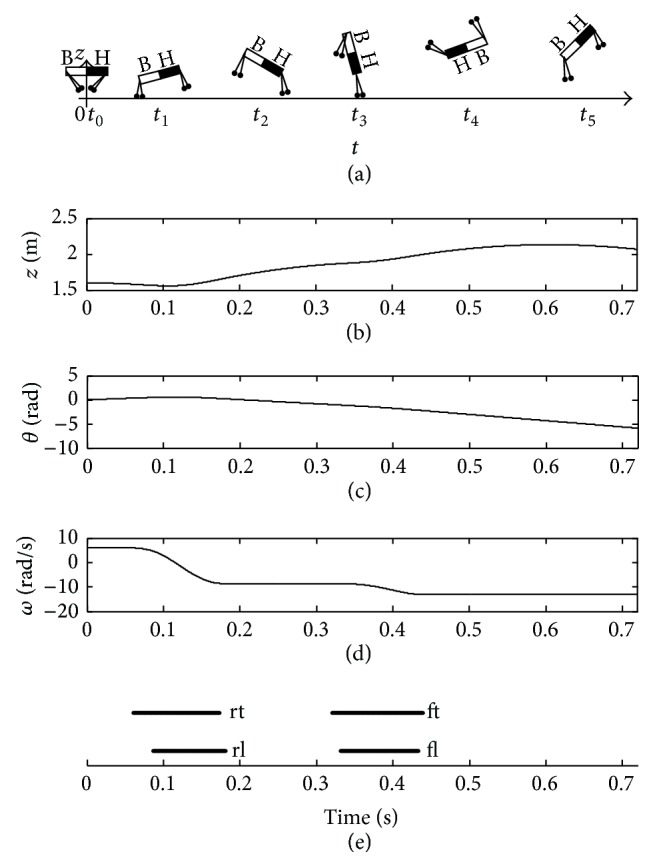
Imbalance posture of the quadruped robot without control.

Figure 11 shows an imbalance posture of the quadruped robot based on the parameters in Table 1 without control. When the initial condition is set as *β*_ft_^td^ = 0.6 rad, *β*_rt_^td^ = 0.5 rad, *d*_*f*_ = 0.1 m, and *d*_*r*_ = 0.5 m, the pitching angle *θ* nearest to zero is at time *t* = 0.2063 s. However, the vertical displacement *z* of COM does not reach the peak at this time. Therefore, after touchdown and liftoff of the front legs, the trunk rotates up to about −2.9 rad at the moment *t*_4_ in Figure 11(c). This will cause a tumbling and even a pulled muscle for the animals.

Therefore, the controlled thrusts of the hind limb are required to stabilize the pitching motion and the height of COM which are considered to be the indicators of stability in sagittal plane. The designed thrusts are given as follows based on (6):

(9) Fc=ΔKfδfll0−lfl+δftl0−lft+ΔKrδrll0−lrl+δrtl0−lrt.

The stiffness variation as the control input is achieved by solving the dynamic equation (1) with the control objectives equation (8). Meanwhile, the galloping stability criterion equation (8) is solved to seek its zero point by a dichotomy algorithm [32, 33]. At last, Δ*K*_*f*_ and Δ*K*_*r*_ are achieved as Δ*K*_*f*_ = −3.9626 × 10^4^ N/m, Δ*K*_*r*_ = −3.4357 × 10^4^ N/m.

**Figure 12:**
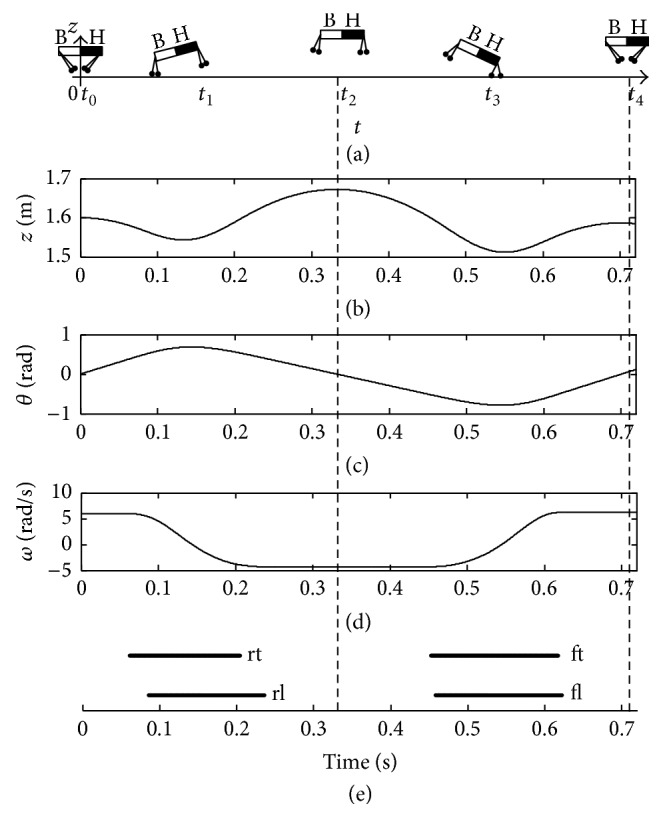
Balance posture of the quadruped robot with control.

**Figure 13:**
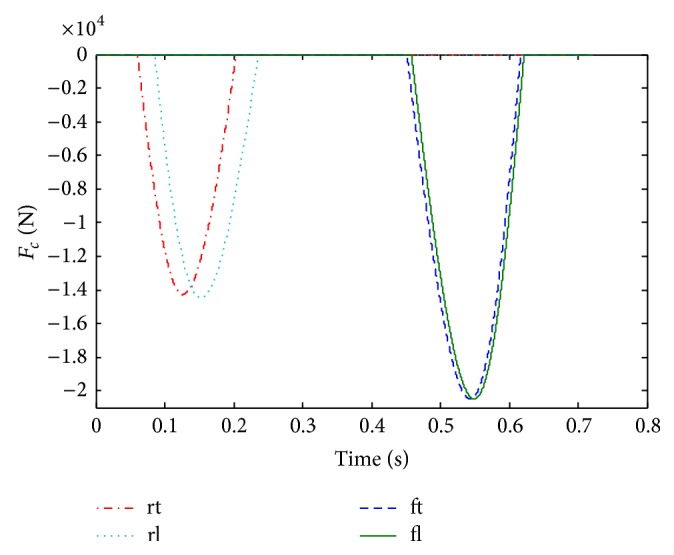
Controlled thrusts.

The control result is presented in Figure 12. With the controlled thrusts, a balance posture of the quadruped robot is achieved. With the stiffness compensation Δ*K*_*r*_, the two rear legs touch down the ground with initial conditions *β*_rt_^td^ = 0.5 rad and *d*_*r*_ = 0.5 m. At the end of the stance phase, the legs lift off the ground with Con_*r*_ = 0 (namely, *ω*_rl_^lo^ *v*_*z*,rl_^lo^ + *gθ*_rl_^lo^ = 0). At the moment *t*_2_, the COM reaches the peak (Figure 12(b)), and, meanwhile, the pitching angle *θ* is zero (Figure 12(c)) during the first flight phase. Furthermore, with the stiffness compensation Δ*K*_*f*_, the front legs touch down the ground with *β*_ft_^td^ = 0.6 rad and *d*_*f*_ = 0.1 m. Then, the front legs lift off the ground with Con_*f*_ = 0, (namely, *ω*_fl_^lo^ *v*_*z*,fl_^lo^ + *gθ*_fl_^lo^ = 0). Finally, at the moment *t*_4_, the trunk reaches the second peak with *v*_*z*_ = 0 and *θ* = 0 during the second flight phase in Figure 12(a). Moreover, the controlled thrusts are calculated and shown in Figure 13; the legend “rt”, “rl”, “ft,” and “fl” indicate the *F*_rt_, *F*_rl_, *F*_ft_, and *F*_fl_, respectively.

## 4. Discussions

### 4.1. Scale Factor of Stability Criterion

As shown in Section 3.1, the stability criterion is applicable for the parameters of a quadruped robot listed in Table 1. In this section, the applicability of the stability criterion is verified for several animals including cat, grey hound, lion, and horse, as their body weights vary from small to large [23–28]. The corresponding parameters are listed in Table 2.

**Figure 14:**
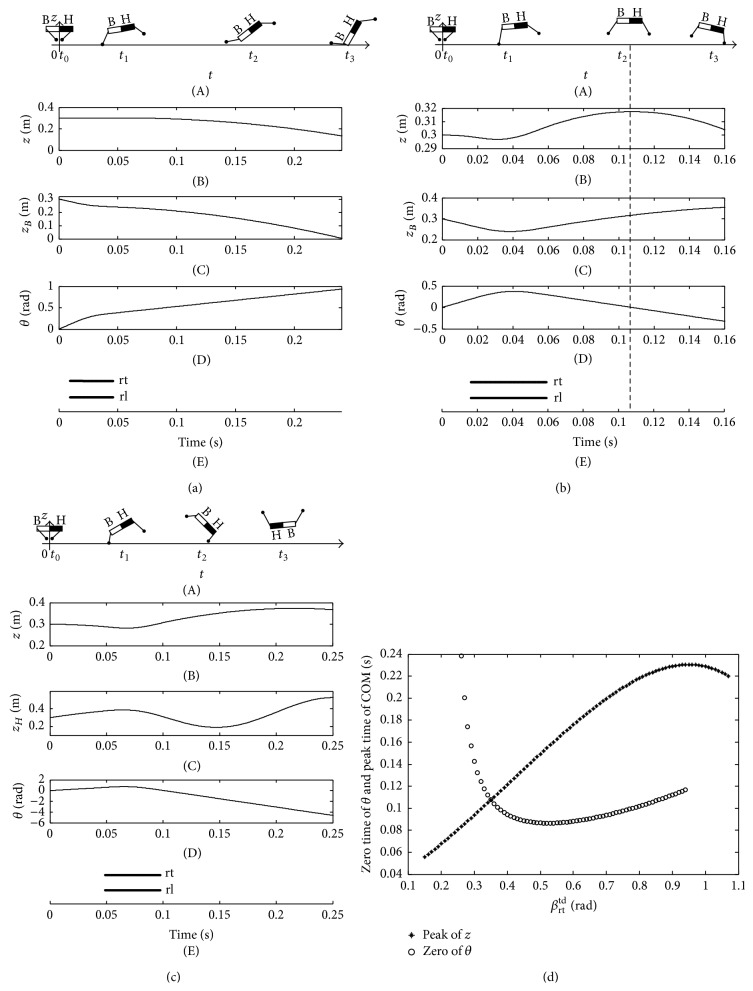
Imbalance and balance postures of cat. (a) The imbalance posture when *β*_rt_^td^ = 0.15 rad; (b) the balance posture when *β*_rt_^td^ = 0.35 rad; (c) the imbalance posture when *β*_rt_^td^ = 0.80 rad; (d) the zero time of pitching angle and the peak time of COM.

As shown in Figure 14, the domestic cat also complies with the stability criterion. When the touchdown angle *β*_rt_^td^ is set as 0.07 rad, at the moment *t*_3_, the bottom of the cat falls down on the ground which is an imbalance posture due to the less thrust provided by the hind limb during stance phase (Figure 14(a)). The balance posture conforming to the stability criterion equation (8) is shown in Figure 14(b) when *β*_rt_^td^ is set as 0.35 rad. In addition, the dashed line indicates the moment when the zero time of pitching angle and peak time of COM are equal. While *β*_rt_^td^ is set as 0.8 rad, the cat somersaults forward during flight phase (Figure 14(c)) for the large thrust of hind limb during stance phase. Meanwhile, Figure 14(d) demonstrates the variation of zero time of pitching angle and peak time of COM with touchdown angle. As stated in Section 3.1, the closer are the two moments which are the zero time of the pitching angle and the peak time of COM, the better is the stability of the torso posture during the flight phase. As a result, the touchdown angle *β*_rt_^td^ at the intersection of the two curves could make the cat form a stable state during flight phase.

**Figure 15:**
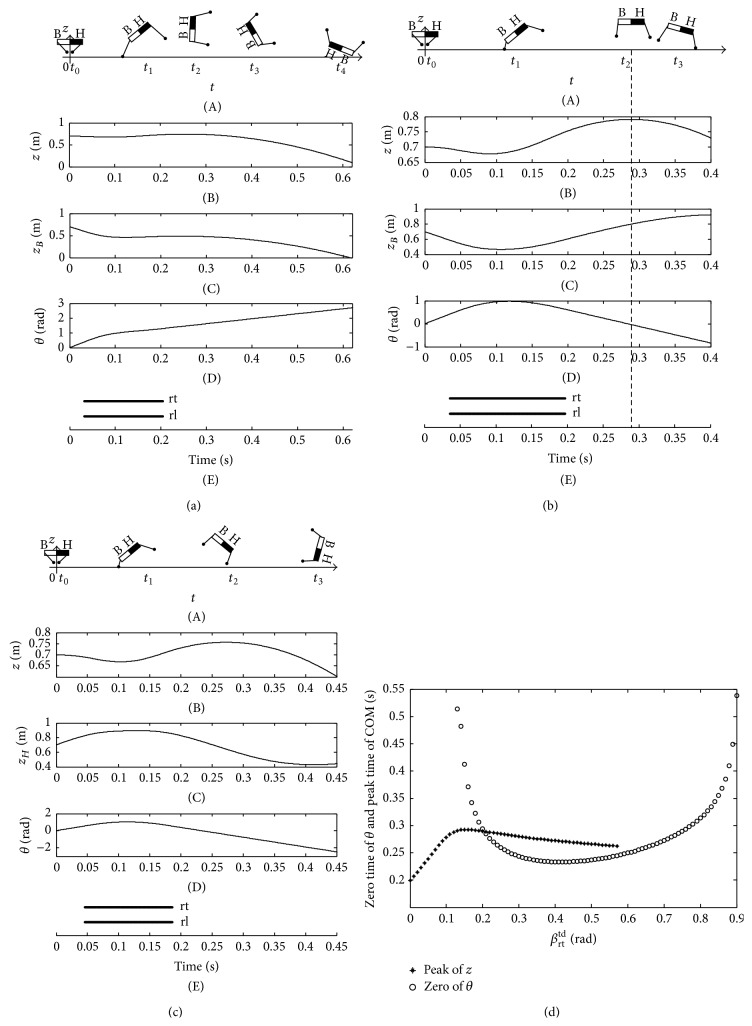
Imbalance and balance postures of greyhound. (a) The imbalance posture when *β*_rt_^td^ = 0.07 rad; (b) the balance posture when *β*_rt_^td^ = 0.21 rad; (c) the imbalance posture when *β*_rt_^td^ = 0.39 rad; (d) the zero time of pitching angle and the peak time of COM.

**Figure 16 fig16:**
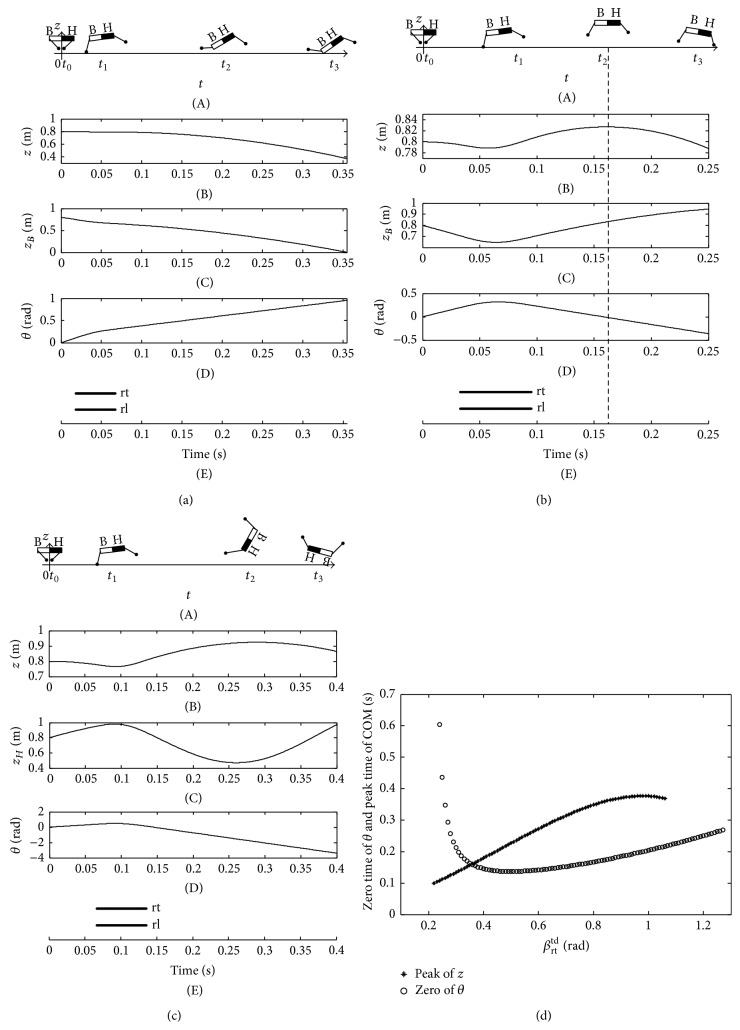
Imbalance and balance postures of lion. (a) The imbalance posture when *β*_rt_^td^ = 0.12 rad; (b) the balance posture when *β*_rt_^td^ = 0.36 rad; (c) the imbalance posture when *β*_rt_^td^ = 0.64 rad; (d) the zero time of pitching angle and the peak time of COM.

The imbalance and balance postures of greyhound, lion, and racehorse are shown in Figures 15 [Fig fig16]~17. All of the tests comply with the stability criterion equation (8). In addition, all the imbalance and balance postures are also similar to the cat. For instance, when the touchdown angle is smaller than the stable touchdown angle (STA) at the intersection of the zero time curve of pitching angle and the peak time curve of COM, the animals will emerge as an overlarge nose-up pitching motion. Thereby, the bottom of the animals will contact the ground. On the contrary, if the touchdown angle is larger than the STA, the overlarge nose-down motion will emerge, and, consequently, the head will contact the ground or the body will generate a clockwise somersault during flight phase. The curves of pitching angle and height of COM vary from animal to animal, although there are same regulations of the tumble for them. Just because of the same regulations of the tumble of greyhound, lion, and racehorse as cat, their imbalance and balance postures will not be elaborated again.

### 4.2. The Applicability of the Stability Criterion for the Animals with Special Body Size

The basset hound and the giraffe are two special animals for the length proportion between leg and torso. The legs of basset hound are much shorter than the torso, while the legs of giraffe are much longer than the torso. The lengths of leg and torso are listed in Table 2. Therefore, we test the applicability of the stability criterion for the two animals in this section.

**Figure 18:**
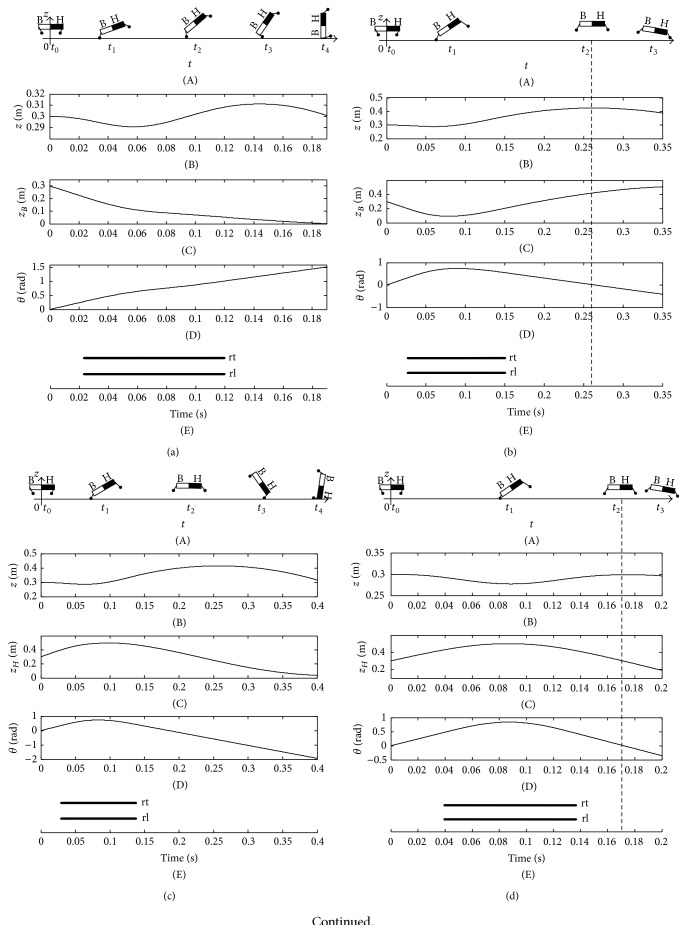
Imbalance and balance postures of basset hound. (a) the imbalance posture when *β*_rt_^td^ = 0.20 rad; (b) the balance posture when *β*_rt_^td^ = 0.40 rad; (c) the imbalance posture when *β*_rt_^td^ = 0.49 rad; (d) the balance posture when *β*_rt_^td^ = 0.78 rad; (e) the zero time of pitching angle and the peak time of COM.

The imbalance and balance postures of basset hound are shown in Figure 18. The overlarge nose-up motion of basset hound emerges which is induced by the small thrust when the touchdown angle is less than the STA. As a result, the bottom of the basset hound will contact the ground when the touchdown angle is set as 0.2 rad (Figure 18(a)). The stable state emerges when the touchdown angle of hind limb is set as 0.4 rad which is equal to the STA. Meanwhile, the zero time of pitching angle that is the peak time of COM is 0.26 s (Figures 18(b) and 18(e)). When the touchdown angle is larger than the STA, the nose-down motion of basset hound emerges. Then, the head contacts the ground when the touchdown angle is set as 0.49 rad (Figure 18(c)). When the touchdown angle is set as 0.78 rad, the zero time of pitching angle and the peak time of COM are very close (Figure 18(e)). According to the stability criterion, the posture of basset hound is almost stable with a touchdown angle of 0.78 rad (Figure 18(d)).

**Figure 19:**
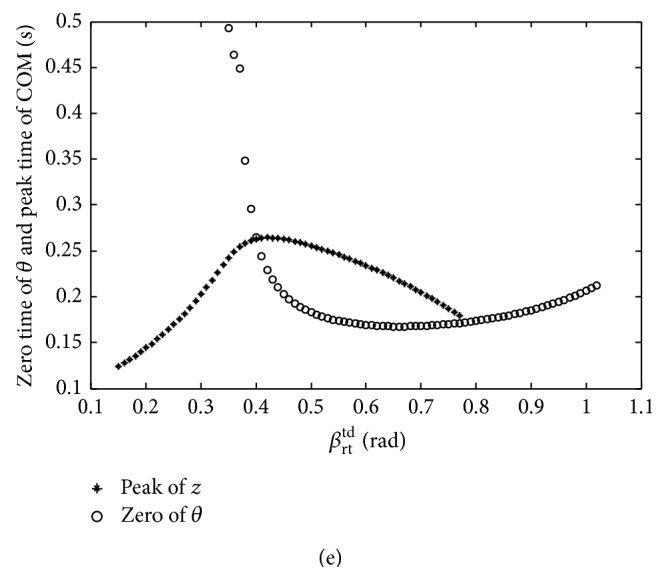
Imbalance and balance postures of giraffe. (a) The imbalance posture when *β*_rt_^td^ = 0.14 rad; (b) the balance posture when *β*_rt_^td^ = 0.34 rad; (c) the imbalance posture when *β*_rt_^td^ = 0.42 rad; (d) the balance posture when *β*_rt_^td^ = 0.50 rad; (e) the imbalance posture when *β*_rt_^td^ = 0.53 rad; (f) the zero time of pitching angle and the peak time of COM.

The imbalance and balance postures of giraffe are shown in Figure 19. There are two intersections between the zero time curve of the pitching angle and peak time curve of COM. Therefore, there are two STAs for the given mechanical parameters and initial values of giraffe listed in Table 2. The first STA is 0.34 rad, and the second one is 0.50 rad. All of the instable touchdown angles are divided into three sections by the two STAs. The peak time of COM is earlier than the zero time of pitching angle when the touchdown angle is less than the first STA or larger than the second STA (Figure 19(f)). As a result, the torso emerges as a positive angle when the giraffe is at the peak time of COM; at last, the backward somersault is generated (Figures 19(a) and 19(e)). The two STAs result in different stable states, and the height of COM and the amplitude of the pitching angle with the second STA are both larger than those with the first STA. That is the larger thrust is exerted by the hind limb with the second STA (Figures 19(b) and 19(d)). The peak time of COM is later than the zero time of pitching angle when the touchdown angle is set inside the range between the first and second STAs. Consequently, the torso emerges as a negative angle when the giraffe is at the peak time of COM. Finally, the imbalance postures with an overlarge nose-down motion are achieved (Figure 19(c)).

### 4.3. The Stability Analysis with the Shift of COM

**Figure 20:**
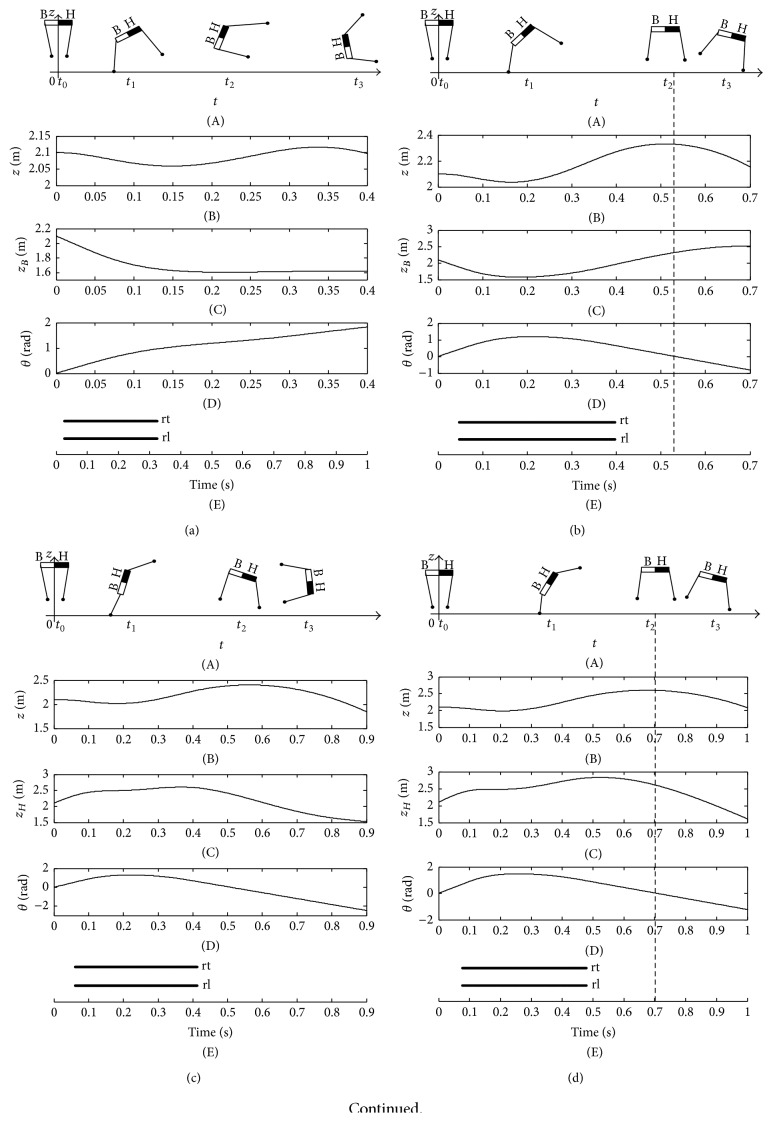
The diagram of force analysis when the carnivores hold a prey in the mouth.

The COM of the carnivore will be shifted when it runs with a prey in its mouth. The running dynamics will be changed with the displacement of COM. So, we analyze the influence of the COM shift on the stability and dynamics of carnivore. The additional mass is in the mouth of the carnivore and varies from 0 to 75 kg. The largest additional mass is twelve percent of the carnivore mass. The ratio between additional weight and carnivore weight can mimic the case that the carnivores hold a prey in the mouth. The simplified force diagram is shown in Figure 20, where *m*_*a*_ indicates the body mass of prey and *m* indicates the body mass of the carnivore. The simulation parameters and the initial values of the carnivore are shown in Table 1.

**Figure 21:**
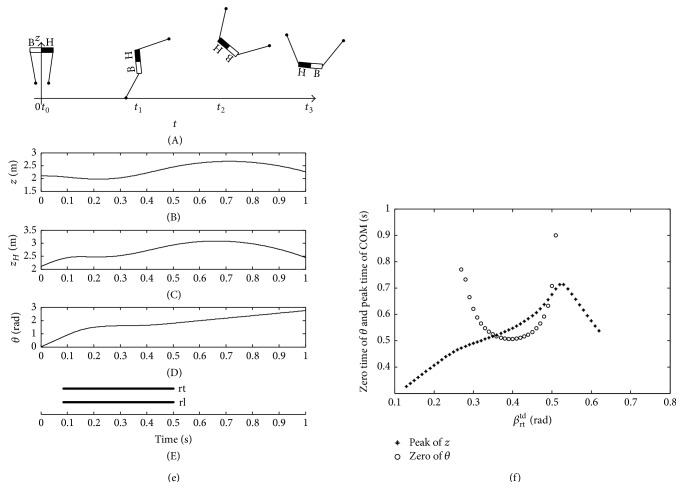
Zero time of pitching angle and peak time of COM. The dark triangles indicate different intersections between the zero time curve of the pitching angle and the peak time curve of COM with different weights of prey.

All of the simulations comply with the stability criterion equation (8) when the additional mass varies from 0 to 75 kg. However, the stable point, that is, the intersection of the zero time curve of pitching angle and the peak time curve of COM, increases almost linearly with the increasing of the prey weight (Figure 21). In other words, the larger the prey weight, the larger the STA and the longer the time needed to achieve the stable state. As a result, larger thrusts provided by the hind limb are needed. However, as shown in Figure 21, the zero time of pitching angle varies from 0.2565 to 0.2632 s, and the time difference of 0.0067 s is a very small interval. The same case emerges in the STA which varies from 0.315 to 0.365 rad. Hence, the angular difference (namely, 0.05 rad) is also a small interval. In conclusion, the influence induced by the COM shift is so little that it could be even neglected.

### 4.4. The Control Strategy Based on Passive Dynamics

The structural parameters of a robot have been fixed when the dynamic model is built; in this case, the stable running could be achieved by adjusting the stiffness and the touchdown angle of leg when the initial conditions are given [21]. When the quadruped robot exposes an imbalance posture with the instable touchdown angle, the leg stiffness is adjusted to compensate the attitude deviation. On the contrary, when the robot exposes an imbalance posture for certain leg stiffness, touchdown angle could be adjusted to compensate the attitude deviation. The latter method is same as the process in which we achieve the stability criterion. At last, both of the control methods comply with the stability criterion equation (8). In this paper, the dynamic equation of the robot is simulated passively which could reveal the inherent dynamic characteristics of the robot. Meanwhile, the control method presented in this paper is also based on the passive dynamics. If a robot runs with the passive stable state, just as the state meets the stability criterion of (8), then it needs a little control action to maintain the stable running [11]. Additionally, the strength of the control action varies monotonously with the deviation degree between the zero time of pitching angle and the peak time of COM. Although the model simulated in our study is simple, the stability criterion is instructive for the design theory and the control strategy of robot.

## 5. Conclusions

In the paper, a stability criterion is presented based on the passive dynamic galloping. First, the dynamical model of the quadruped robot is constructed on the basis of the energy conservation law. Through the mathematical model, the imbalance and balance postures of a passive quadruped robot are analyzed. Then, a necessary and sufficient balance condition that is the stability criterion is attained. In addition, the validity of the stability criterion is verified against cat, greyhound, lion, and racehorse with the body weight varying from small to large. Besides, the stability criterion is applicable to the basset hound and giraffe, whose legs are much shorter or much longer than the torso. Furthermore, the applicability of the stability criterion is verified when the carnivore holds a prey in its mouth. The results support the conclusion that the influence of the COM shift on the stability criterion is almost negligible. Next, the controlled thrust is obtained with an optimization dichotomy algorithm for seeking the zero point of the stability criterion when the animal exposes imbalance postures. Finally, the imbalance postures of a galloping gait are stabilized with the controlled thrust by adjusting the stiffness of the legs. The validity of the stability criterion and the control method are verified by the controlled results.

## Figures and Tables

**Figure 17:**
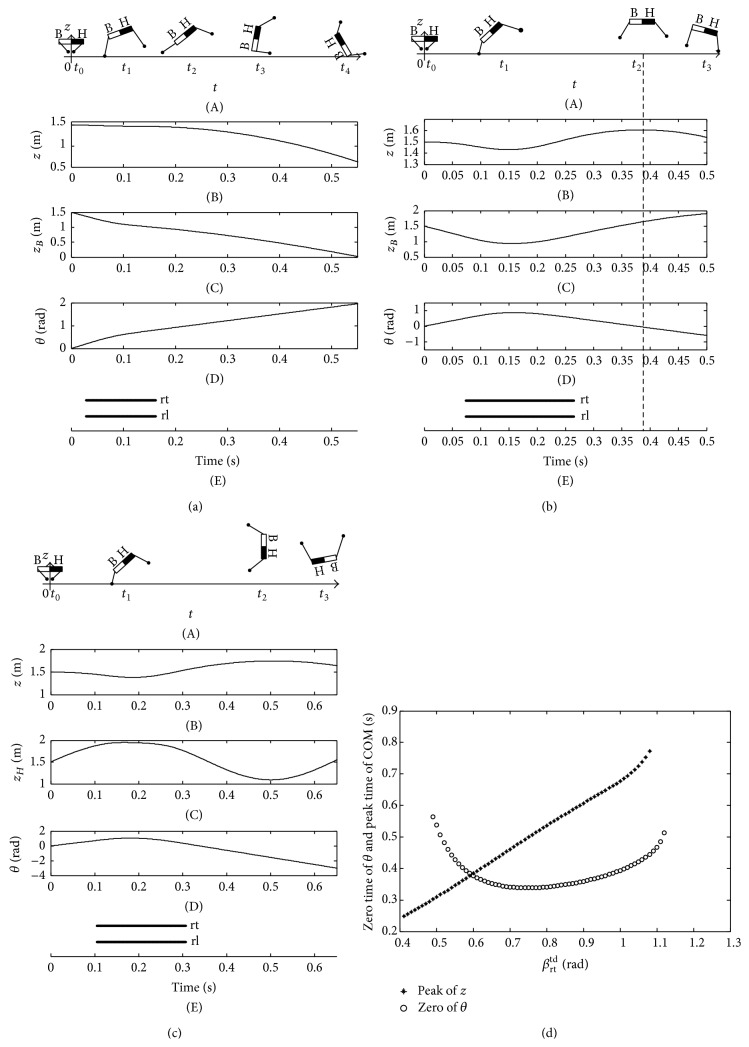
Imbalance and balance postures of racehorse. (a) The imbalance posture when *β*_rt_^td^ = 0.21 rad; (b) the balance posture when *β*_rt_^td^ = 0.60 rad; (c) the imbalance posture when *β*_rt_^td^ = 0.76 rad; (d) the zero time of pitching angle and the peak time of COM.

**Table 1:** System parameters and initial conditions.

Variable	Value
*m* (kg)	640
*K* (N/m)	62200
*l*_0_ (m)	1.5
*l*_*b*_ (m)	0.75
*J* (kgm^2^)	240
*v*_*x*0_ (m/s)	11
*ω*_0_ (rad/s)	6
*z*_0_ (m)	1.6
*x*_0_ (m)	0
*v*_*z*0_ (m/s)	0
*θ*_0_ (rad)	0
*d*_*f*_ (m)	0.6
*d*_*r*_ (m)	0.6
*β*_ft_^td^ (rad)	0.3540
*β*_rt_^td^ (rad)	0.2122
*F*_*i*_ (N)	0
*τ*_*i*_ (Nm)	0

**Table 2:** Parameters of animals and the initial values of simulation [23–28].

Variable	Cat	Greyhound	Lion	Racehorse	Basset hound	Giraffe
*m* (kg)	3.7	35	167	900	25	1200
*K* (N/m)	3200	7000	62200	62200	7000	62200
*l*_0_ (m)	0.285	0.6	0.755	1.4	0.22	2
*l*_*b*_ (m)	0.16	0.26	0.45	0.65	0.3	0.5
*v*_*x*0_ (m/s)	5	1	9	8	1	3
*ω*_0_ (rad/s)	12	12	6	7	12	9
*z*_0_ (m)	0.3	0.7	0.8	1.5	0.3	2.1
*x*_0_ (m)	0	0	0	0	0	0
*v*_*z*0_ (m/s)	0	0	0	0	0	0
*θ*_0_ (rad)	0	0	0	0	0	0

## References

[1] Raibert M. H., Brown H. B., Chepponis M. (1984). Experiments in blance with a 3D one-legged hopping machine. *International Journal of Robotics Research*.

[2] Geyer H., Seyfarth A., Blickhan R. (2005). Spring-mass running: simple approximate solution and application to gait stability. *Journal of Theoretical Biology*.

[3] Seyfarth A., Geyer H., Herr H. (2003). Swing-leg retraction: a simple control model for stable running. *Journal of Experimental Biology*.

[4] Blum Y., Lipfert S. W., Rummel J., Seyfarth A. (2010). Swing leg control in human running. *Bioinspiration & Biomimetics*.

[5] Qing T., Rong X., Jian C. (2009). Tip over avoidance control for biped robot. *Robotica*.

[6] Deng Q., Wang S. G., Liang Q. H., Mo J. Q. (2010). The effect of body pitching on leg-spring behavior in quadruped running. *Journal of Bionic Engineering*.

[7] Wei X., Wang C., Long Y., Wang S. (2014). Pitch angular velocity dynamics property in transverse galloping pattern from the passive dynamic perspective. *Proceedings of the Institution of Mechanical Engineers, Part C: Journal of Mechanical Engineering Science*.

[8] Nanua P., Waldron K. J. (1995). Energy comparison between trot, bound, and gallop using a simple model. *Journal of Biomechanical Engineering*.

[9] Poulakakis I., Smith J. A., Buehler M. (2005). Modeling and experiments of untethered quadrupedal running with a bounding gait: the scout II robot. *The International Journal of Robotics Research*.

[10] Poulakakis I., Papadopoulos E., Buehler M. (2006). On the stability of the passive dynamics of quadrupedal running with a bounding gait. *International Journal of Robotics Research*.

[11] Chatzakos P., Papadopoulos E. (2009). Bio-inspired design of electrically-driven bounding quadrupeds via parametric analysis. *Mechanism and Machine Theory*.

[12] Smith J. A., Poulakakis I., Trentini M., Sharf I. (2010). Bounding with active wheels and liftoff angle velocity adjustment. *International Journal of Robotics Research*.

[13] Marhefka D. W., Orin D. E., Schmiedeier J. P., Waldron K. J. (2003). Intelligent control of quadruped gallops. *IEEE/ASME Transactions on Mechatronics*.

[14] Deng Q., Wang S., Xu W., Mo J., Liang Q. (2012). Quasi passive bounding of a quadruped model with articulated spine. *Mechanism and Machine Theory*.

[15] Wei X., Long Y., Wang C., Wang S. (2014). Rotary galloping with a lock–unlock elastic spinal joint. *Proceedings of the Institution of Mechanical Engineers, Part C: Journal of Mechanical Engineering Science*.

[16] Berkemeier M. D. (1998). Modeling the dynamics of quadrupedal running. *The International Journal of Robotics Research*.

[17] Zhao Q., Sumioka H., Nakajima K., Yu X., Pfeifer R. (2014). Spine as an engine: effect of spine morphology on spine-driven quadruped locomotion. *Advanced Robotics*.

[18] Krasny D. P., Orin D. E. (2010). Evolution of a 3d gallop in a quadrupedal model with biological characteristics. *Journal of Intelligent and Robotic Systems: Theory and Applications*.

[19] Hornby G. S., Takamura S., Yamamoto T., Fujita M. (2005). Autonomous evolution of dynamic gaits with two quadruped robots. *IEEE Transactions on Robotics*.

[20] Chae K. G., Park J. H. (2009). Trajectory optimization with GA and control for quadruped robots. *Journal of Mechanical Science and Technology*.

[21] Seyfarth A., Geyer H., Günther M., Blickhan R. (2002). A movement criterion for running. *Journal of Biomechanics*.

[22] Herr H. M., McMahon T. A. (2001). A galloping horse model. *The International Journal of Robotics Research*.

[23] Kashiwamura F., Avgaandorj A., Furumura K. (2001). Relationships among body size, conformation, and racing performance in banei draft racehorses. *Journal of Equine Science*.

[24] Mitchell G., Van Sittert S. J., Skinner J. D. (2009). Sexual selection is not the origin of long necks in giraffes. *Journal of Zoology*.

[25] Toma S., Colombo S., Cornegliani L. (2008). Efficacy and tolerability of once-daily cephalexin in canine superficial pyoderma: an open controlled study. *Journal of Small Animal Practice*.

[26] Cecchi F., Carlini G., Ciani E., Bramante A., Ciampolini R. (2011). A survey on morphological traits of Basset Hound dogs raised in Italy. *Journal of Life Sciences*.

[27] Day L. M., Jayne B. C. (2007). Interspecific scaling of the morphology and posture of the limbs during the locomotion of cats (Felidae). *Journal of Experimental Biology*.

[28] Hudson P. E., Corr S. A., Wilson A. M. (2012). High speed galloping in the cheetah (*Acinonyx jubatus*) and the racing greyhound (*Canis familiaris*): spatio-temporal and kinetic characteristics. *The Journal of Experimental Biology*.

[29] Cao Q., Poulakakis I. (2013). Quadrupedal bounding with a segmented flexible torso: passive stability and feedback control. *Bioinspiration & Biomimetics*.

[30] Kapti A. O., Yucenur M. S. (2006). Design and control of an active artificial knee joint. *Mechanism and Machine Theory*.

[31] François C., Samson C. (1998). A new approach to the control of the planar one-legged hopper. *The International Journal of Robotics Research*.

[32] Ferrer J. (2007). Zeroes of real polynomials on C(K) spaces. *Journal of Mathematical Analysis and Applications*.

[33] Bento A. J., Silva C. M. (2013). Generalized nonuniform dichotomies and local stable manifolds. *Journal of Dynamics and Differential Equations*.

